# Transanal total mesorectal excision after incomplete endoscopic submucosal dissection for early-stage low rectal cancer: A small case series

**DOI:** 10.1016/j.ijscr.2022.107590

**Published:** 2022-09-02

**Authors:** Mamoru Miyasaka, Shuji Kitashiro, Shunichi Okushiba, Tetsuya Sumiyoshi, Hiroko Takeda, Satoshi Hirano

**Affiliations:** a Department of Surgery, Tonan Hospital, Japan; b Department of Gastroenterology, Tonan Hospital, Japan; c Department of Pathology, Tonan Hospital, Japan; d Department of Gastroenterological Surgery II, Hokkaido University Faculty of Medicine Hokkaido, Japan

**Keywords:** ESD, endoscopic submucosal dissection, TaTME, transanal total mesorectal excision, DRM, distal resection margin, LAR, low anterior resection, Additional resection, Early stage, Endoscopic submucosal dissection, Low rectal cancer, Transanal total mesorectal excision, Case series

## Abstract

Endoscopic submucosal dissection (ESD) for colorectal cancer is challenging but is gradually being performed worldwide. It is less invasive than surgical resection and can be performed on lesions in which malignancy cannot be diagnosed. In low rectal cancers, changes such as scarring after ESD may make it challenging to preserve the anus when additional surgical resection is required. Transanal total mesorectal excision (TaTME) is a novel surgical technique involving transanal endoscopic manipulation. It is useful for lesions in the deep pelvis near the anus. Herein, we report six cases of TaTME after ESD for early-stage low rectal cancer that resulted in incomplete resection. As a representative case, a 77-year-old female was referred to our hospital, and colonoscopy revealed low rectal cancer. ESD was performed, and the pathological diagnosis was an invasion of the submucosal layer and microscopic lymphovascular invasion. We performed an additional laparoscopic low anterior resection with TaTME. Lymph node metastasis was observed, and the final diagnosis was pT1b, pN1a, pStage IIIa, and R0. In other cases, the anus can also be preserved, and the distal margin can be secured. TaTME enabled anal preservation without being affected by the ESD scars. It is considered useful for additional resection after ESD of low rectal cancer.

## Highlights

• Additional resection after ESD for low rectal cancer is difficult.
• TaTME has become an attractive surgical approach to achieve an accurate DRM.
• TaTME has made it possible to secure the DRM, even after ESD for low rectal cancer.

## 1 Introduction

Endoscopic submucosal dissection (ESD) is a novel treatment for benign colorectal tumors and early cancers that achieves *en bloc* mucosal resection with wider margins [1]. ESD is currently a widely used treatment with advances in techniques and equipment [2]. While diagnostic treatment is possible if additional resection is required (e.g., positive vertical margin or submucosal invasion in a malignant tumor), ESD scars make surgery challenging. Resection and anastomosis at the scar site may lead to anastomotic leakage and stenosis. In addition, anal preservation may be challenging because of the proper distal resection margin (DRM) from the ESD scar.

Low anterior resection (LAR) for low rectal cancer is challenging for lesions located closer to the anus. The narrow and deep pelvis has a poor field of view in open surgery and is restricted by fixed trocar positions and straight laparoscopic instruments, even during laparoscopic surgery. In recent years, transanal total mesorectal excision (TaTME) has become an attractive minimally invasive surgery. Performing a transanal “bottom-up” surgical approach can achieve an accurate DRM with adequate visualization during surgery [3]. Herein, we describe six cases of TaTME after ESD for early-stage low rectal cancer.

## 2 Case report

### 2.1 Patient & method

We performed additional resections in patients with low rectal cancer who could not undergo complete resection by ESD. Patients were retrospectively enrolled at a single center (Tonan Hospital) between January 2019 and December 2021, excluding patients aged ≥90 years and American Society of Anesthesiologists physical status classification (ASA-PS) ≥ 3. This study is registered with the ResearchRegistry and the unique identifying number is: researchregistry8205 (https://www.researchregistry.com/browse-the-registry#home/). This case series has been reported in line with the PROCESS Guideline [4]. Curative resection could not be achieved, defined as satisfying all the following criteria based on the Japanese Society for Cancer of the Colon and Rectum guidelines 2019 for the treatment of colorectal cancer [5]: negative vertical and lateral margins, depth of submucosal invasion of <1000 μm, negative vascular invasion, and negative budding. In post-ESD rectal cancer, surgery is mainly performed using only a laparoscopic approach for high or middle rectal cancer. For low rectal cancers below the peritoneal reflection, the TaTME approach was selected, as shown in this case series. All surgeries were performed by a team of experienced surgeons and there was no conversion to laparotomy or other changes during surgery. The patient characteristics are summarized in Table 1. TaTME is a transanal approach that creates pneumoperirectum with carbon dioxide [3]. Direct viewing with an endoscope allows the transanal pursestring suture to be performed below the tumor, ensuring that an adequate oncological distal margin is achieved.

**Table 1:** Patient characteristics after ESD.

Case	Age	Sex	BMI	Location of lesion^a^	Histological type	Tumor size (mm)	Depth of invasion (μm)	Vascular invasion^b^	Budding^c^
1	72	F	24.8	Rb, 7 cm	tub1	28 × 26	1400	ly1, v0	BD1
2	45	F	26.1	Rb, 2.5 cm	tub1-muc	35 × 27	4000	ly0, v1	BD2
3	57	M	22.6	Rb, 6 cm	tub1-tub2	33 × 27	1000	ly1, v1	BD1
4	77	F	21.2	Rb, 7 cm	tub1	28 × 26	3000	ly1, v1	BD2
5	81	M	22.4	Rb, 7 cm	tub1 > tub2	25 × 20	4300	ly0, v1	BD1
6	55	M	19.1	Rb, 4 cm	tub2 > tub1, por1	27 × 21	7000	ly0, v1	BD1

a Distance from the anal verge to the lesion.

b ly: lymphatic vessels invasion, v; vein invasion.

c BD; budding grade.

### 2.2 A representative case

**Fig. 1:**
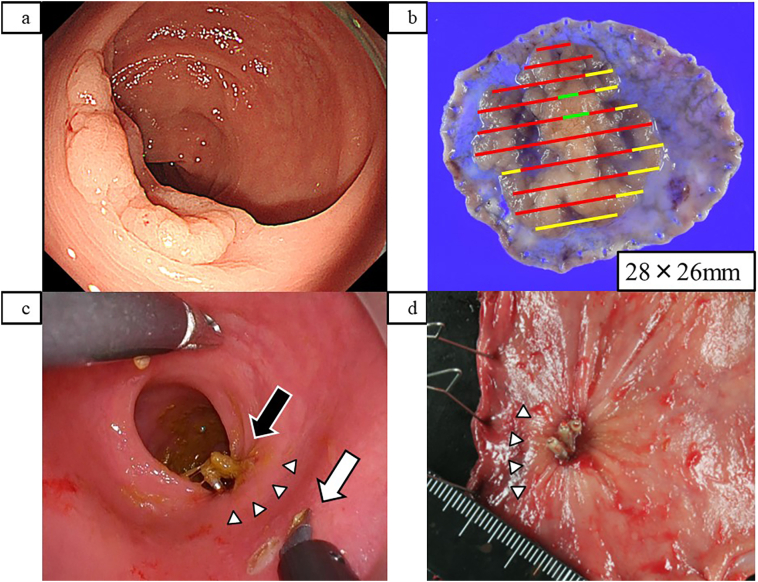
(a) Colonoscopic image A 0-IIa granular-type lesion with a laterally spreading tumor in the lower rectum. (b) Mapping image of the postoperative pathology. The horizontal margins were negative, and submucosal invasion was localized to a small area (red line: tub1 adenocarcinoma in mucosa, green line: submucosal invasive cancer, yellow line: adenoma). (c) View of the transanal approach. The clip on the endoscopic submucosal dissection (ESD) scar is checked and an incision with an appropriate distal resection margin is made. (d) Anal wedge of the resected rectal specimen. Clips were placed on the ESD scar. Black arrow, ESD scar and clips; white triangles, boundaries of changes in the ESD scars on the mucosal surface; white arrow, incision line. (For interpretation of the references to color in this figure legend, the reader is referred to the web version of this article.)

Herein, we present a case representative of our series (Case 4 in Table 1). A 77-year-old female patient was referred to our hospital for further evaluation and treatment of a positive fecal occult blood test. Colonoscopy revealed a 0-IIa granular-type lesion with a laterally spreading tumor in the lower rectum (Fig. 1a). The lesion was diagnosed as a group 5 adenocarcinoma by biopsy. ESD was performed, and the tumor was resected *en bloc*. The time required for ESD was 122 min. The tumor measured 28 × 26 mm, and the lateral margin was negative. However, the pathological diagnosis was well-differentiated adenocarcinoma with an invasion of 3000 μm into the submucosal layer, ly1a, v1a, and budding grade 2 (Fig. 1b). Additional surgery was performed to ensure proper lymph node dissection. One month after ESD, laparoscopic LAR with TaTME was performed. The ESD scar was confirmed using the transanal approach during surgery, and the anal side was dissected at an appropriate distance (Fig. 1c). The operative time was 112 min, and the estimated blood loss was <5 cc. The postoperative pathological diagnosis revealed no residual tumor or metastasis of adenocarcinoma to the lymph nodes (Fig. 1d). The final diagnosis was tub1 adenocarcinoma, pT1b (SM2), pN1a, pStage IIIa, R0. The patient was discharged 13 days after surgery with no complications. With regard to postoperative complications in this case series, complications of Clavien-Dindo classification ≥ III were observed in only one patient [6]. It was small bowel perforation caused by laparoscopic manipulation.

Table 2 shows the surgical results and pathological findings after additional resection for post-ESD low rectal cancer in this case series. Lymph node metastasis was observed in three of the six cases by additional resection. Even in obese and male patients, it was possible to confirm the ESD scar and secure the DRM using a transanal procedure.

**Table 2:** Surgical results and final pathological diagnosis.

Case	Age	Sex	Surgical procedure^a^	Operative time (min)	Blood loss (cc)	Lymph node metastasis	Distal margin (mm)
1	72	F	LAR	168	30	pN0	20
2	45	F	ISR	215	<5	pN2a	10 + α^b^
3	57	M	LAR	221	35	pN1a	14 + α^b^
4	77	F	LAR	112	<5	pN1a	20
5	81	M	LAR	186	<5	pN0	25
6	55	M	SLAR	188	<5	pN0	15 + α^b^

ISR: intersphincteric resection, LAR: low anterior resection, SLAR: super low anterior resection, TaTME: transanal total mesolectal excision.

a All cases performed by laparoscopic surgery with TaTME.

b Includes stump of a few mm with an single stapling technique.

## 3 Discussion

Surgical resection for rectal cancer has historically been recognized as the gold standard based on the principles of total mesorectal excision [7]. However, despite advances in techniques and surgical staplers, surgical resection has an inherent complication rate around 10 % [8]. LAR can be technically challenging, especially in obese and male patients. Therefore, one could argue that surgery may be too invasive for early-stage low rectal cancers.

ESD has been developed as a technique for treating gastric lesions. Colorectal ESD is challenging to perform because the colorectal wall is thinner than the stomach wall and has a higher risk of perforation. Several techniques and equipment have been developed to improve safety [2]. Comparing laparoscopic resection and ESD for colorectal lesions, it has also been reported that the former has complications such as wound infection, ileus, and urinary tract infection [9]. Treatment with ESD, which is also useful for diagnosis, is worth performing for low rectal lesions.

**Fig. 2:**
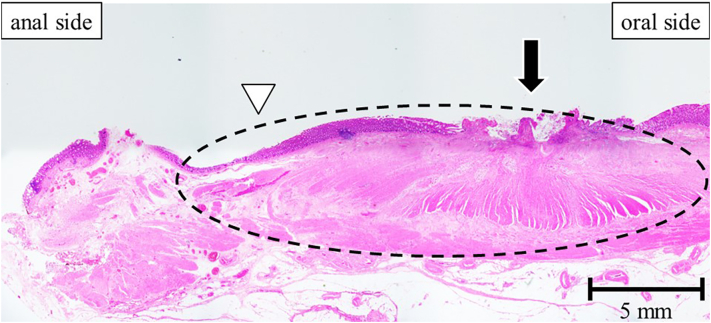
Pathological examination revealed fibrotic changes extended from the endoscopic submucosal dissection (ESD) scar to the muscularis propria on the anal side. Black arrow, site of the ESD scar with the clip; white triangle, boundaries of changes in the ESD scars on the mucosal surface; black dotted line, range where the fibrosis extends to the muscularis propria on the anal side.

The problem with additional resection after ESD for low rectal cancer is scarring. Histologically, post-ESD fibrotic changes may extend vertically across the muscularis propria to the fascia propria of the rectum and horizontally from the ESD scar on the mucosal surface to the anal side of the muscularis propria. Even in our representative case (Case 4 in Table 1), fibrotic changes were observed in the muscularis propria on the anal side of the ESD scar (Fig. 2c). Resection and anastomosis at the ESD scar site may increase the risk of complications such as anastomotic leakage and stenosis. Oncologically, resection at an appropriate distance from the ESD scar is important. The risk of positive margins has been reported to be significant after colorectal surgery, particularly for low and anterior rectal tumors [10]. TaTME makes it possible to directly confirm the lesions (tumors or ESD scars) using the transanal approach. TaTME helps obtain high-quality specimens and lower rates of positive DRM and circumferential resection margins, which can affect patient prognosis [11]. Although not studied in post-ESD cases, TaTME has been reported to have fewer complications in challenging cases [12]. In our case series, preserving the anus while maintaining the distal margin was possible.

This study has some limitations. First, the number of cases was small, and only a single institution was involved in the study. Second, the oncological outcomes are unknown because no long-term observations have been made. However, the patient in Case 1 survived with no recurrence for 3 years with the longest observation period, and no recurrence occurred in any of the cases. Third, at our institution, TaTME is performed by two teams, the transanal and abdominal teams, as we believe that surgery will take longer if performed by one team.

## 4 Conclusions

TaTME has made it possible to secure the distal margin, even after ESD for low rectal cancer. Anal preservation was possible without interference caused by the ESD scars. Further studies are needed to confirm these findings.

## Sources of funding

This research did not receive any specific grant from funding agencies in the public, commercial, or not-for-profit sectors.

## Provenance and peer review

Not commissioned, externally peer-reviewed.

## Ethical approval

This is an observational study. The Tonan Hospital Research Ethics Committee has confirmed that no ethical approval is required.

## Consent

The subjects provided informed consent, and patient anonymity was preserved.

## Registration of research studies

This study is registered with the ResearchRegistry and the unique identifying number is: researchregistry8205 (https://www.researchregistry.com/browse-the-registry#home/).

## Guarantor

Mamoru Miyasaka

## CRediT authorship contribution statement

Conception and study design: M. Miyasaka, S. Kitashiro

Acquisition of data: M. Miyasaka, S. Kitashiro, S. Okushiba, T. Sumiyoshi, H. Takeda

Analysis and/or interpretation of data: M. Miyasaka, S. Kitashiro

Drafting the manuscript: M. Miyasaka, S. Kitashiro

Revising the manuscript critically for important intellectual content: M. Miyasaka, S. Kitashiro, S. Hirano

Approval of the version of the manuscript to be published (the names of all authors must be listed): M. Miyasaka, S. Kitashiro, S. Okushiba, T. Sumiyoshi, H. Takeda, S. Hirano

## Declaration of competing interest

There are no conflicts of interest to declare.
